# Management of Residual Hearing with Cartilage Conduction Hearing Aid after Lateral Temporal Bone Resection: Our Institutional Experience

**DOI:** 10.3390/audiolres11020024

**Published:** 2021-06-09

**Authors:** Noritaka Komune, Yoshie Higashino, Kazuha Ishikawa, Tomoko Tabuki, Shogo Masuda, Kensuke Koike, Takahiro Hongo, Kuniaki Sato, Ryutaro Uchi, Masaru Miyazaki, Ryo Shimamoto, Nana Akagi Tsuchihashi, Ryunosuke Kogo, Teppei Noda, Nozomu Matsumoto, Takashi Nakagawa

**Affiliations:** 1 Department of Otorhinolaryngology, Graduate School of Medical Sciences, Kyushu University, Fukuoka 812-8582, Japan; y-hgsn@qent.med.kyushu-u.ac.jp (Y.H.); horikiri@med.kyushu-u.ac.jp (K.I.); tabuki@med.kyushu-u.ac.jp (T.T.); shogo.masuda117@gmail.com (S.M.); kensuke11242000@gmail.com (K.K.); orihakat.ognoh@gmail.com (T.H.); basement.kuni13@gmail.com (K.S.); urentcello@gmail.com (R.U.); nanaakagi0707@yahoo.co.jp (N.A.T.); ryukogo@gmail.com (R.K.); teppei@med.kyushu-u.ac.jp (T.N.); matsumoto.nozomu.297@m.kyushu-u.ac.jp (N.M.); nakataka@med.kyushu-u.ac.jp (T.N.); 2 Department of Otorhinolaryngology, Fukuoka University Hospital and School of Medicine, Fukuoka 812-8582, Japan; masarumiyazaki@fukuoka-u.ac.jp; 3 Department of Plastic Surgery, Graduate School of Medical Sciences, Kyushu University, Fukuoka 812-8582, Japan; say.hello.to.ryo@gmail.com

**Keywords:** temporal bone resection, hearing management, cartilage conduction hearing aid

## Abstract

Background: There is no guideline for hearing compensation after temporal bone resection. This study aimed to retrospectively analyze surgical cases with reconstruction for hearing preservation after temporal bone malignancy resection and propose a new alternative to compensate for hearing loss. Methods: We retrospectively reviewed the medical records of 30 patients who underwent lateral temporal bone surgery for temporal bone malignancy at our institution and examined their hearing abilities after surgery. Result: The hearing outcomes of patients with an external auditory meatus reconstruction varied widely. The mean postoperative air–bone gap at 0.5, 1, 2, and 4 kHz ranged from 22.5 dB to 71.25 dB. On the other hand, the average difference between the aided sound field thresholds with cartilage conduction hearing aid and bone conduction thresholds at 0.5, 1, 2, and 4 kHz ranged from −3.75 to 41.25. More closely located auricular cartilage and temporal bone resulted in smaller differences between the aided sound field and bone conduction thresholds. Conclusions: There is still room for improvement of surgical techniques for reconstruction of the auditory meatus to preserve hearing after temporal bone resection. The cartilage conduction hearing aid may provide non-invasive postoperative hearing compensation after lateral temporal bone resection.

## 1. Introduction

Malignant tumors of the temporal bone are rare with an extremely low incidence rate [1,2]. The most common histological type is squamous cell carcinoma, followed by adenoid cystic carcinoma. Currently, the establishment of clinical evidence is slow due to the rarity of this entity. In the existing literature, negative margin resection has been recognized to some extent as the standard of treatment. However, there is currently no global consensus on the treatment protocol.

Additionally, each facility may have various treatment strategies to compensate for hearing loss after temporal bone resection. Patients with temporal bone malignancies are often provided with treatment options that result in hearing loss. Hearing loss leads to the deterioration of patients’ quality of life. To compensate, hearing improvement after surgery is desirable, but there is no standard protocol or guideline on this issue.

It has been shown that negative margin resection for temporal bone malignancies provides excellent long-term tumor-free survival. While the use of hearing preservation surgery with auditory canal reconstruction and tympanoplasty after temporal bone resection has recently been reported, surgical results remain under discussion and in need of improvement. Morita et al. reported favorable results in eight cases of auditory canal reconstruction using split-thickness skin grafts for surgically treated early temporal bone malignancies [3]. However, few reports have detailed postoperative hearing results [3,4].

Nishimura’s group first introduced cartilage conduction hearing in clinical practice. A cartilage conduction hearing aid (CCHA) includes both the cartilage–bone sound pathway and the cartilage–air and direct air pathway. This small and non-invasive device was considered as an option for hearing compensation after lateral temporal bone surgery [5,6,7,8,9,10].

In this report, to discuss options for hearing compensation after lateral temporal bone resection (LTBR), we report the postoperative hearing progression of cases with external auditory canal reconstruction after lateral temporal bone resection (LTBR) and the results of our examination of the effectiveness of the CCHA after LTBR.

## 2. Material and Methods

### 2.1. Patient Selection

A retrospective review of the patients treated at the Department of Otorhinolaryngology, Head and Neck Surgery at the Kyushu University Hospital from January 1993 to July 2020 was performed. A total of 181 patients were treated for temporal bone-related malignancies. A total of 161 cases of malignancies originated from the temporal bone. LTBR cases with postoperative hearing compensation were selected for this review. The final dataset included nine patients who underwent LTBR with the reconstruction of the external auditory meatus and tympanoplasty. Furthermore, we obtained audiometric data from 16 cases aided with CCHAs. Approval from the ethics review committee of Kyushu University Hospital (permit no. 29–43) was obtained.

### 2.2. Treatment Strategy for Temporal Bone Squamous Cell Carcinoma at Our Institute

All patients with temporal bone squamous cell carcinoma were treated with LTBR. When a postoperative pathological examination revealed a positive resection margin or if it was highly suspected intraoperatively, postoperative chemoradiotherapy was added. When the tumor was considered resectable with free negative margins on preoperative computed tomography (CT) and magnetic resonance imaging (MRI) scans, reconstruction of the external auditory canal with a free flap was planned for the patient undergoing hearing-preserving surgery.

### 2.3. Audiometric Data

Audiometry with a pure-tone audiometer (AA-76, AA-78, AA-79; Rion, Kokubunji, Japan) was conducted in a soundproof booth by experienced audiologists. Pure-tone thresholds were measured at 0.125, 0.25, 0.5, 1, 2, 4, and 8 kHz frequencies for air conduction and at 0.25, 0.5, 1, 2, 4 kHz for bone conduction with masking as appropriate. The results of both preoperative and postoperative hearing thresholds are included in our dataset. The hearing level was evaluated based on pure-tone audiograms as a follow-up to postoperative hearing levels in patients with auditory canal reconstruction. Pure-tone air and bone conduction thresholds averages were obtained. For pure-tone averages, the thresholds measured were 0.5, 1, 2, and 4 kHz. Air-bone gaps (ABGs) were calculated using air and bone conduction averages from the same test. To test the hearing level in patients that underwent surgery with the bone–cartilage anchoring technique, ipsilateral pure-tone hearing thresholds were tested while the patients wore commercial CCHAs (HB-J1CC, Rion) with appropriate masking for the contralateral side. We calculated and averaged the difference between the aided sound field thresholds and bone conduction thresholds at 0.5, 1, 2, and 4 kHz, which is referred to as “aided ABG.”

### 2.4. Image Analysis

An axial image of CT was used to measure the closest distance between the auricular cartilage and temporal bone after surgery.

## 3. Results

### 3.1. Patient Profile

Our study included 30 patients that underwent LTBR, among which nine cases underwent the reconstruction of the external auditory meatus and tympanoplasty and 12 cases underwent the closure of the external auditory meatus. Five out of 12 cases underwent LTBR with the bone–cartilage anchoring technique to establish firm contact between the cartilage and the temporal bone. We obtained audiometric data from 16 cases aided with CCHAs after surgery. Pathology, clinical T stage (based on the modified Pittsburgh classification), sex, age, affected side, type of surgical approach, type of free flap for reconstruction, operation time, surgeon, resection margin examination, adjuvant radiotherapy, and aided ABG are summarized in Table 1. In cases 11 and 13, tumor invasion of the resected margin was highly suspected intraoperatively; for this reason, postoperative radiotherapy was added, although surgical margins were reported as free of carcinoma. In case 15 and 20, postoperative radiotherapy was added because of the extranodal extension. Case 8 and 10 purchased the hearing aid after surgery. The rest of the patients decided not to purchase the hearing aid yet, because their hearing level on the contralateral side was still adequate.

### 3.2. Reconstruction of the External Auditory Meatus with a Free Flap and Hearing Outcome

**Figure 1:**
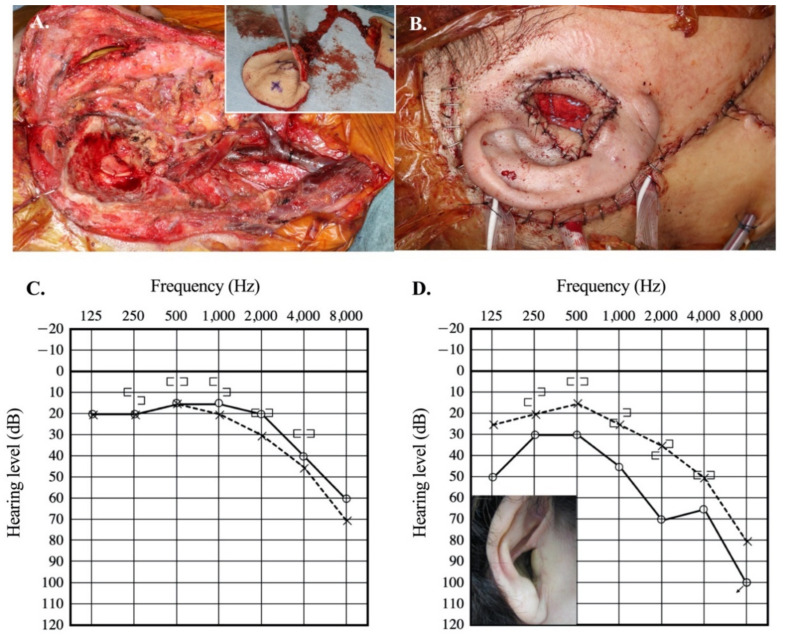
Reconstruction of the external auditory canal after lateral temporal bone resection. (**A**) Surgical view of completed lateral temporal bone resection. An inset shows the harvested free anterolateral thigh flap, which is rolled to create the external auditory meatus. (**B**) Final view after reconstruction of the external auditory meatus. (**C**) Preoperative pure-tone audiometry (Case 1) (**D**) Postoperative pure-tone audiometry one year after surgery (Case 1). Inset shows the reconstructed auditory canal in Case 1.

The surgical steps for the reconstruction of the external auditory meatus with a free flap are shown in Figure 1. After the en bloc LTBR was done, an anterolateral thigh flap (Cases 1–5, 7 and 9) or groin flap (Cases 6 and 8) with a vascular pedicle was elevated. The skin island flap for the tympanic membrane and auditory meatus was prepared and rolled (Figure 1A). For the tympanic membrane, the subcutaneous tissue was removed to produce a thin layer of vascularized skin. The rolled flap was placed into the temporal bone defect. At the same time, the skin of the tympanic membrane was attached to the bony or cartilage columella on the stapes head (type III tympanoplasty) (Figure 1B). Preoperative and 1-year postoperative pure-tone audiometry results and the reconstructed external auditory meatus in a representative case are shown in Figure 1C,D.

**Figure 2:**
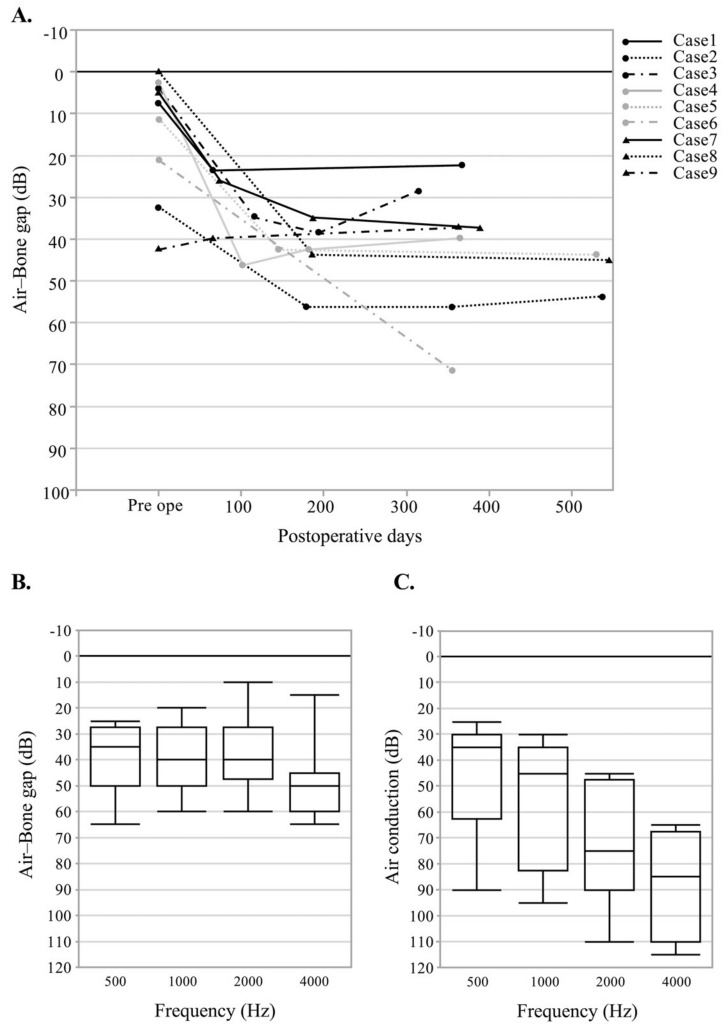
Postoperative hearing level of nine cases with reconstruction of the auditory canal with a free flap. (**A**) Hearing outcome of air–bone gap after surgery in nine patients. (**B**) Air–bone gap by frequency after surgery. (**C**) Air conduction level by frequency after surgery. The horizontal line within the box represents the median sample value. Box boundaries represent the 1st and 3rd quartiles. Whiskers extend from quartiles to the minimum/maximum data point.

The postoperative follow-up for hearing levels was reviewed in all nine patients with external auditory canal reconstruction. Mean postoperative air–bone gap varied from 22.5 dB to 71.25 dB (Figure 2A). At 2 kHz, the postoperative ABG was at a minimum and varied from 10 dB to 60 dB (Figure 2B).

Postoperative air conduction level also varied from 25 dB to 90 dB at 0.5 Hz, from 30 dB to 95 dB at 1 kHz, from 45 dB to 110 dB at 2 kHz, and from 65 dB to 115 dB at 4 kHz (Figure 2C). The auditory meatus was preserved in eight out of nine patients. In case 6, the volume of the free flap was too great to maintain the structure of the auditory canal and resulted in stenosis of the auditory meatus, which ensued in a mean postoperative ABG of 71.25 dB. This patient was in the process of planning an additional surgery to reduce the volume of the flap and conserve the external auditory meatus.

### 3.3. Effectiveness of the CCHA

**Figure 3:**
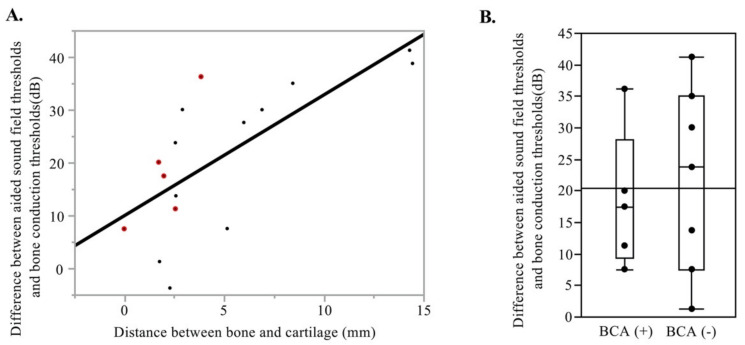
Effectiveness of the cartilage conduction hearing aid in cases after lateral temporal bone resection. (**A**) The relationship defining the distance between the auricular cartilage and the temporal bone and the difference between the aided sound field and bone conduction thresholds in 16 cases with the cartilage conduction hearing aids. Red dots show cases with BCA. (**B**) Difference between the aided sound field and bone conduction thresholds in 12 cases without external auditory meatus reconstruction. BCA; bone-cartilage anchoring.

We examined the audiometric data of 16 patients wearing the CCHAs. In four cases with and 12 cases without external auditory reconstruction, we obtained audiometric data postoperatively using the CCHAs. The results showed that the average difference between the aided sound field thresholds and bone conduction thresholds at 0.5, 1, 2, and 4 kHz ranged from −3.75 to 41.25. There was a moderate correlation between the distance between the auricular cartilage and the temporal bone around the triangular fossa and the postoperative difference between the aided sound field thresholds and bone conduction thresholds. Here, the closer the distance, the smaller the difference (*p* = 0.0021 R2 = 0.503; Figure 3A). When comparing patients who underwent intraoperative cartilage–bone anchoring with those who did not, treated patients showed lower mean values but with no statistical significance (Figure 3B).

### 3.4. Bone-Cartilage Anchoring Technique

**Figure 4:**
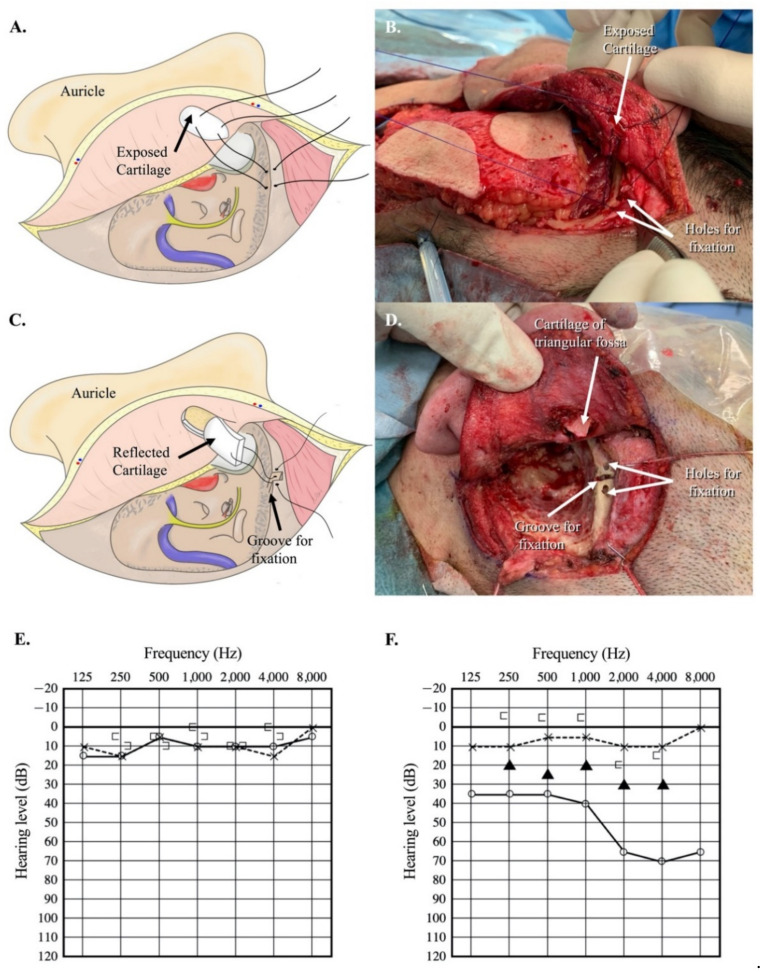
Bone–cartilage anchoring technique (BCA). (**A**) Auricular cartilage was anchored to the temporal bone with a 3-0 PDS suture (Type 1). (**B**) Surgical view of the Type 1 bone–cartilage anchoring technique (Case 13). (**C**) The auricular cartilage was inserted into the created groove of the temporal bone and fixed with a 3-0 PDS suture (Type 2). (**D**) Surgical view of the type 2 bone–cartilage anchoring technique (Case 12). (**E**) Preoperative pure-tone audiometry (Case 10). (**F**) Postoperative pure-tone audiometry of case 10 after the bone–cartilage anchoring technique. The black triangle shows the hearing level with a cartilage conduction hearing aid at a sound field with adequate masking on the contralateral side.

**Figure 5:**
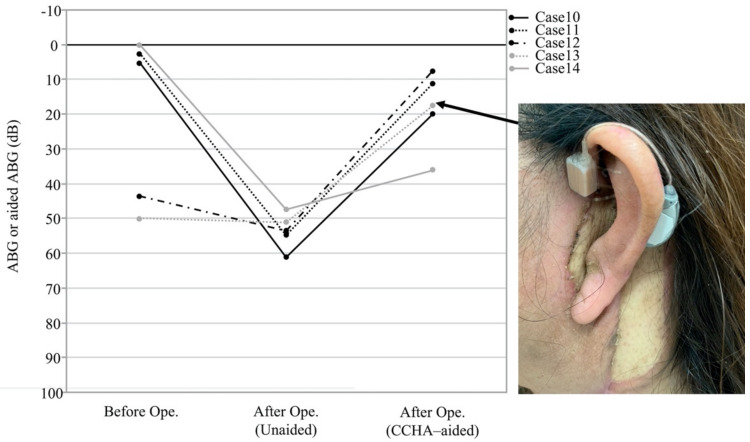
The outcome of the air–bone gap after the bone–cartilage anchoring technique surgery. An inset shows the auricular wearing the cartilage conduction hearing aid (Case 13). The CCHA transducer was fixed to the anterior root of the helix with double-sided tape. The difference between the aided sound field and bone conduction thresholds was represented as an air–bone gap under the CCHA-aided state after surgery. CCHA, cartilage conduction hearing aid; Ope., operation.

At our institution, we devised an intraoperative method to establish contact between the cartilage and the bone that increased the effectiveness of the CCHAs in five cases. After LTBR, the cartilage of the triangular fossa is exposed from the wound surface. Two types of anchoring were proposed. The first option is to fix the surface of the triangular fossa cartilage to the temporal bone (Figure 4A,B). The second option is to fix the reflected cartilage of the triangular fossa to the created bony groove at the temporal bone (Figure 4C,D). Preoperative and postoperative hearing levels of a representative patient (Case 3) are shown in Figure 4E,F. Postoperative pure-tone audiometry revealed an apparent conductive hearing loss on the ipsilateral side (Figure 4F). CCHA use improved the hearing level on the ipsilateral side, and the average of the aided sound field threshold with appropriate masking on the contralateral side resulted in 26.25 dB (Figure 4F). For the five patients treated with the bone–cartilage anchoring technique, we calculated the difference between the aided sound field and bone conduction thresholds postoperatively. The average difference at 0.5, 1, 2, and 4 kHz was less than 25 dB postoperatively for the four patients (Figure 5). In case 14, the average difference at 0.5, 1, 2, and 4 kHz was 36.25, but the distance between the auricular cartilage and the temporal bone was largest among cases using the bone–cartilage anchoring technique (Figure 5).

## 4. Discussion

The only currently considered standard of treatment for temporal bone malignancies in the world is en bloc and negative margin resection [11,12,13]. Previous reports have shown that patients with a negative margin resection have an excellent long-term prognosis in both early and advanced stages. LTBR and subtotal temporal bone resection (STBR) have been widely used for en bloc resection of temporal bone malignancies. STBR includes the resection of the inner ear structure, making it impossible to conserve hearing postoperatively. By contrast, LTBR preserves the inner ear structure, but this treatment will result in conductive hearing loss. Various reconstruction methods have been reported for postoperative temporal bone defects [14,15,16,17]. However, only a few reports have considered hearing preservation by combining tympanoplasty and external auditory canal reconstruction [3,4,18]. In 2016, the UK Guideline for Management of Lateral Skull Base Cancer was published. The guideline confirmed that a hearing deficit is an inevitable outcome of temporal bone resection but did not provide any reconstruction options to preserve hearing. The guideline did, however, describe rehabilitation for total hearing loss. Total conductive hearing loss can be rehabilitated through an osseointegrated bone-anchored hearing aid (BAHA) or a bilateral contralateral routing of signals aid [19].

Both complete resection of the tumor and hearing compensation after surgery are necessary to maintain the quality of life of patients. To date, there are four options to maintain or correct ipsilateral hearing: (1) reconstruction with a local flap, (2) reconstruction with a free pedicled flap, (3) middle ear implant or BAHA, and (4) bone conduction hearing aid. As of 1 April 2021, neither the middle ear implant or the BAHA for unilateral hearing deficit were covered by health insurance in Japan. Therefore, patients must choose from the other three options. Each option has advantages and disadvantages, as shown in Table 2.

In 2013, Iida et al. reported the reconstruction of the external auditory canal with a free flap after LTBR [18]. To reconstruct the external auditory canal, a relatively thin myocutaneous flap is needed. This limits the harvest site for a free flap to the forearm, groin, or anterolateral thigh. It is relatively easy to collect a flap thin enough to make the auditory canal from the forearm; however, because this is an exposed area, harvesting a flap may result in a cosmetic problem. A flap harvested from the groin can also be thin, but a long feeding blood vessel is difficult to collect in this area. The anterolateral thigh flap is thicker but has the advantage of being a less exposed area and having long feeding vessels, which convey a higher degree of freedom in anastomosis construction. In our institution, the anterolateral thigh flap is preferred for reconstruction (seven out of nine patients). When a tumor is considered resectable with negative surgical margins on preoperative CT and MRI scans, reconstruction of the external auditory canal with a free flap is considered if the patient wants to undergo hearing-preserving surgery. To avoid the risk of stenosis of the auditory canal, delayed wound healing, and complications from postoperative radiotherapy, a well-vascularized free flap is used for the reconstruction of the external auditory canal (if required) [20]. In our series, the subcutaneous tissue from the flap was removed to produce a thin layer of vascularized skin, which was used to reconstruct the tympanic membrane.

It is well known that, in general, the volume of the free flap decreases gradually after surgery. However, not all cases involving free flap reconstruction follow the same clinical course. We encountered a case in which the volume of the free flap was conserved, resulting in stenosis of the reconstructed external auditory meatus (Case 6). Thus, the surgeon should preoperatively explain the possibility of a staged surgery to reduce the flap volume if necessary. Additionally, there is a possibility of deviation of the columella, resulting in worsening conductive hearing loss. Furthermore, hearing results depend on the patient. Only two patients achieved <30 dB of a mean postoperative ABG. Considering that hearing results largely rely on both patient factors and surgeon skills, there is a significant amount of room for improving this surgical procedure.

Adjuvant radiotherapy can cause osteoradionecrosis, the elevation of the sensorineural hearing thresholds, and radiation-induced otitis media and externa (dermatitis). Thus, LTBR with external auditory canal reconstruction and tympanoplasty is recommended only in cases with a high possibility of a margin-free resection based on the preoperative radiological evaluation. However, we cannot predict the result of a postoperative histopathological examination of all surgical cases. In our series, four out of nine patients had a positive margin resection, although the preoperative radiological assessment seemed to predict negative surgical margins. To prevent postoperative osteoradionecrosis of the temporal bone, the best option is to fill the surgical defect with well-vascularized tissue. Considering these aspects, an alternative method for postoperative hearing compensation is needed for cases with a high possibility of adjuvant radiotherapy.

Because a reconstructed auditory canal does not have a natural self-cleaning mechanism, cleaning the reconstructed auditory canal after surgery should also be considered. A preoperative explanation should be given to patients on how the reconstructed ear canal should be cleaned regularly for the rest of their life. On the other hand, strong contact with the skin and the pressure exerted against the cranial bone from the bone conduction hearing aids can cause skin erosion and pain. To overcome these disadvantages, we used a CCHA in a case with LTBR to maintain hearing postoperatively. In Japan, CCHAs have become commercially available [5,6,7,8,9,10]. This type of hearing aid is small and requires less pressure on the contact area. If the CCHA can compensate for postoperative hearing disturbances, the patient can avoid skin complications in the reconstructed ear canal. Furthermore, we considered that the desired hearing level could be achieved regardless of the surgical result of the reconstruction.

The hearing results of the patient with a CCHA after LTBR implied that the distance separating the auricular cartilage and the temporal bone is a potential factor for improving the effectiveness of sound transfer using the CCHAs after LTBR. With the cartilage anchored to the temporal bone, as shown in Figure 4, CCHAs may effectively transfer sound after LTBR. This technique is very simple, and every surgeon can provide the same quality of care. Results of the average difference between the aided sound field thresholds and bone conduction thresholds at 0.5, 1, 2 and 4 kHz in five cases with the cartilage conduction hearing devices are shown in Figure 5. We found a satisfactory result of less than 25 dB of mean postoperative difference between the aided sound field and bone conduction thresholds in four out of five cases (Figure 5).

Nishimura’s group reported several advantages of CCHAs [5,6,7,8,9,10]. A CCHA includes both the cartilage–bone sound pathway and the cartilage air and direct air pathways. Furthermore, Morimoto et al. reported that fibrotic tissue connected to the ossicles provides an additional pathway, which is termed the fibrotic tissue pathway [21]. They mentioned that a substantial connection of occluding fibrotic tissue with the ossicles implied the presence of a fibrotic tissue pathway. We found a substantial connection of occluding fibrotic tissue with the ossicles in 10 out of 16 cases. These cases may use both the fibrotic tissue pathway and the cartilage–bone pathway. It has the advantage of aiding patients with outer ear disorders, such as atresia of the external auditory canal. This makes the approach suitable for cases that involve temporal bone resection. However, disconnection between the cartilage and bone may result in sound transmission disturbance. Our technique overcame this issue by compensating for hearing loss postoperatively with the CCHA. Furthermore, patients that use bone conduction hearing aids often suffer from pain and discomfort due to the strong contact that the aid has with the skin and the pressure that it exerts against the cranial bone. Cartilage conduction does not require strong and sustained pressure on the skin. Thus, we first introduced the CCHA in postoperative cases of LTBR. In these cases, the auditory canal was closed and they did not need to clean the auditory canal. Postoperative hearing in these cases was satisfactory. The bone–cartilage anchoring technique is a simple procedure to establish contact between the auricular cartilage and the temporal bone, which may improve sound transfer in patients with a CCHA after LTBR. It could be one effective option to compensate hearing ability after LTBR.

A limitation of this study was its small sample size. Further studies are warranted to validate our preliminary data. However, based on the present data, we predict that CCHAs will be introduced to more patients treated using the bone–cartilage anchoring technique.

## 5. Conclusions

This paper presented the hearing outcomes and options for hearing compensation after LTBR. The information obtained from our review can be extrapolated to offer guidance on reconstruction for these patients. Surgeons should consider hearing compensation for surgical cases of temporal bone malignancies as well as curative surgical resection.

## Figures and Tables

**Table 1:** Case profiles.

	#	Pathology	cT	Sex	Age	Side	Approach	Reconstruction	Ope Time	Surgeon		Margin	PORT	(Gy)	BCA	Aided ABG (dB)
										ENT	Plastic					
With EAC reconstruction	1	w-m SCC	1	F	74	R	LTBR	ALT	9 h 22 min	NM	SY	−	−	0	−	-
	2	w-m SCC	1	M	62	R	LTBR	ALT	10 h 26 min	TN/NM	KK	−	−	0	−	-
	3	w SCC	1	F	61	R	LTBR	ALT	10 h 19 min	NM	RS/SY	+	+	60	−	-
	4	w SCC	1	F	78	R	LTBR	ALT	6 h 41 min	NM	SY	−	−	0	−	-
	5	w SCC	4	F	72	R	LTBR	ALT	10 h 41 min	NK	RS	+	+	60	−	30
	6	w-m SCC	4	F	66	R	LTBR	Groin	9 h 00 min	NK	SY	−	−	0	−	38.75
	7	ACC	1	F	83	R	LTBR	ALT	9 h 39 min	NM	SY	−	−	0	−	-
	8	ACC	2	M	58	R	LTBR	Groin	9 h 53 min	TNo/NK	HK	+	+	60	−	27.5
	9	ACC	4	F	76	L	LTBR	ALT	14 h 19 min	TNo/NK	RS	+	+	70	−	−3.75
Without EAC reconstruction	10	w SCC	4	F	33	R	LTBR	ALT	17 h 57 min	TNo/NK	KI	+	+	60	+	20
	11	w-m SCC	2	F	67	R	LTBR	ALT	12 h 44 min	NK	RS/KI	−	+	50	+	11.25
	12	w SCC	2	M	56	L	LTBR	PAT	5 h 32 min	NK	RS	−	−	0	+	7.5
	13	w SCC	2	F	55	L	LTBR	ALT	8 h 56 min	NK	SY	−	+	60	+	17.5
	14	w SCC	4	F	66	L	LTBR	ALT	13 h 8 min	NK	RS	−	−	0	+	36.25
	15	w-p SCC	2	F	69	L	LTBR	TM	7 h 30 min	NK	NK	−	+	60	−	30
	16	w SCC	4	F	48	L	LTBR	ALT	15 h 38 min	NK	SF	−	−	0	−	41.25
	17	w SCC	1	F	68	L	LTBR	ALT	9 h 36 min	NK	HK	−	−	0	−	35
	18	w SCC	4	F	60	R	LTBR	ALT	10 h 21 min	NK	KI	−	−	0	−	23.75
	19	w SCC	3	F	66	R	LTBR	TM	6 h 31 min	NK	NK	−	−	0	−	13.75
	20	w SCC	4	F	71	R	LTBR	ALT	12 h 48 min	NK	CO	−	+	60	−	7.5
	21	w SCC	2	M	66	L	LTBR	PAT	6 h 51 min	NK	YI	−	−	0	−	1.25

The difference between the aided sound field thresholds and bone conduction thresholds at 0.5, 1, 2, and 4 kHz were calculated and averaged, which is referred to as “aided ABG.” ACC, adenoid cystic carcinoma; ALT, anterolateral thigh; BCA, bone-cartilage anchoring technique; CCHA, cartilage conduction hearing aid; EAC, external auditory canal; LTBR, lateral temporal bone resection; PAT, perifascial areolar tissue; PORT, postoperative radiotherapy; SCC, squamous cell carcinoma; w, well differentiated; w-m, well to moderately differentiated; w-p, well to poorly differentiated.

**Table 2:** Hearing compensation after temporal bone surgery.

**Hearing Loss Compensation after Temporal Bone Resection**		
	Advantages	Disadvantages
**Free Flap Reconstruction**		
	1. The possibility to maintain the hearing level without hearing aid	1. The postoperative volume of the flap can’t be predicted preoperatively. Thusly, surgeon should explain the staged surgery to reduce the volume of the flap to maintain the external ear canal if necessary
	2. The easy detection of the tumor recurrence through the canal	2. Need to clean the auditory canal regularly
	3. The possibility to use the hearing aid with ear mold	3. The possibility of recurrent tumor exposure
		4. The ear mold is needed to be renewed depends on the volume of the flap
**Local Flap Reconstruction**		
	1. Less invasive	1. Deterioration of the conductive hearing loss and otorrhea, caused by Stenosis, Contracture, chronic infection and bone exposure
	2. The possibility to maintain the hearing level without hearing aid	2. Delay wound healing
	3. The easy detection of the tumor recurrence through the canal	3. Dual local flaps and skin grafting are often needed
	4. The possibility to use the hearing aid with ear mold	4. Need to clean the auditory canal regularly
**Bone Conductive Haring Aid (No ear canal)**		
	1. No need of postoperative clean-up of the auditory canal	1. Strong contact to the skin and pressure against the cranial bone of bone-conductive hearing aid cause the skin erosion and patient’s pain.
	2. The maintain the hearing level with hearing aid	2. Residual and recurrent disease need to be detected only by radiological examination.
	3. Prevent the tumor exposure when the tumor is recurrent	3. Expensive (purchasing expense, repair cost, etc.)
**Cartilage Conductive Hearing (No ear canal)**		
	1. No need of postoperative clean-up of the auditory canal	1. Residual and recurrent disease need to be detected only by radiological examination.
	2. Hearing aid is small and right	2. Expensive (purchasing expense, repair cost, etc.)
	3. Prevent the tumor exposure when the tumor is recurrent	
	4. No strong pressure to the skin and cranial bone	

## Data Availability

The authors confirm that the data supporting the findings of this study are available within the article.
